# Plans, Habits, and Theory of Mind

**DOI:** 10.1371/journal.pone.0162246

**Published:** 2016-09-01

**Authors:** Samuel J. Gershman, Tobias Gerstenberg, Chris L. Baker, Fiery A. Cushman

**Affiliations:** 1 Department of Psychology, Harvard University, Cambridge, MA, United States of America; 2 Department of Brain and Cognitive Sciences, Massachusetts Institute of Technology, Cambridge, MA, United States of America; Ecole Normale Superieure, FRANCE

## Abstract

Human success and even survival depends on our ability to predict what others will do by guessing what they are thinking. If I accelerate, will he yield? If I propose, will she accept? If I confess, will they forgive? Psychologists call this capacity “theory of mind.” According to current theories, we solve this problem by assuming that others are rational actors. That is, we assume that others design and execute efficient plans to achieve their goals, given their knowledge. But if this view is correct, then our theory of mind is startlingly incomplete. Human action is not always a product of rational planning, and we would be mistaken to always interpret others’ behaviors as such. A wealth of evidence indicates that we often act habitually—a form of behavioral control that depends not on rational planning, but rather on a history of reinforcement. We aim to test whether the human theory of mind includes a theory of habitual action and to assess when and how it is deployed. In a series of studies, we show that human theory of mind is sensitive to factors influencing the balance between habitual and planned behavior.

## Introduction

How do humans make sense of each other’s behavior? Decades of research in psychology converge on a basic answer to this question about our theory of mind. Across cultures, and from an early age, humans describe, explain and predict each other’s behavior based on the *principle of rational action* [1–6]. According to this principle, people assume that others’ behaviors are governed by plans, and that these plans are chosen to maximize expected subjective utility. Computational models that formalize the principle of rational action as planning (e.g., [7–14]) have proven to be extremely successful in predicting human judgments about the beliefs, desires, plans and behaviors of other people.

This model of mental state inference is simple, elegant and well-supported, yet it posits that humans employ a dramatically incomplete theory of the actual causes of behavior. We are not always rational actors: Humans routinely act not on the basis of plans, but instead on the basis of habit, reflex or instinct [15, 16]. The distinction between planning and habitual action is fundamental across the behavioral sciences and is supported by current research into the neuroscience of learning and choice [17, 18]. It also captures some important elements of a longstanding interest in psychology in the distinction between automatic and controlled behavior [19–23].

Here, we begin to characterize how the concept of habitual action is situated in the folk theory of mind. Of course, an initial question is whether people ordinarily possess a “folk theory of habits”. Although our experiments reveal the answer, it is not a matter of much suspense. In everyday contexts we routinely describe behavior as habitual; clearly the concept occupies some role in our theory of mind, however neglected in prior empirical research.

Rather, our studies are motivated principally by two aims. First we assess whether people attribute habits preferentially in circumstances that actually favor habitual control. To this end, we present participants with scenarios and ask them to make predictions about another agent’s actions, while manipulating several factors that have been experimentally demonstrated to affect the relative strength of habits in guiding actual human behavior (e.g., repetition, consistency, cognitive load). If the folk theory of mind resembles contemporary computational accounts of decision making, we expect action predictions to deviate from the principle of rational action under conditions that favor habitual control.

Second, we assess whether the attribution of habitual control has significant downstream influences on social cognition. We adopt moral judgment as a case study. People assign less blame to harmful actions performed unintentionally. (As Justice Holmes famously quipped, “Even a dog knows the difference between being kicked and being stepped on”). Consequently, psychologists have devoted great effort to characterizing the role of theory of mind in moral judgment [24, 25]. Nearly all of this research assumes that the relevant mental state attributions are of beliefs, desires, plans and goals—in other words, constituents of the principle of rational action. Far less attention has been devoted to the moral evaluation of habitual action.

We contrast two hypotheses about the moral evaluation of habitual action. An important motive for morally evaluating social partners is to motivate them to change their behavior, for instance by punishing their harmful actions [26, 27]. Like plans, habits are sensitive to reinforcement, and so punishing habitual actions can lead to behavior change. Thus, people may hold others morally responsible for their habitual actions. Yet, some evidence suggests that habitual or automatic behaviors are judged to be beyond a person’s control [28], and independent evidence suggests that people are not blamed for uncontrollable actions [29]. Thus, people may assign less blame and punishment to harmful actions that are produced by habits than to harmful actions that are produced by planning.

Before proceeding, a caveat is in order. To say that people sometimes act habitually does not entail that they are irrational actors, or that their behavior is not planning-based [30, 31]. There are two senses in which habits embody principles of rationality. First, as mandated by computational theories of reinforcement learning, habits lead towards reward maximizing patterns of behavior provided an agent has sufficient experience in an environment, and assuming that reward-relevant aspects of the environment don’t change spontaneously. Habits are by definition inflexible and therefore will exhibit failure modes in cases where planning will succeed. This occurs, for instance, when an agent knows about changes to their environment but has not yet directly experienced those changes. This shortcoming notwithstanding, there is a second dimension of rationality to habitual action: Although it is relatively inflexible, it is computationally cheap to deploy during online decision-making. In many contexts, the benefits of efficiency outweigh the costs of inflexibility, embodying a rational trade-off [32]. Thus, our claim is not that habits are irrational *per se* but that they can in certain circumstances lead to locally irrational behavior—a violation of expected value maximization conditioned on the agent’s current beliefs and desires. Our experiments investigate whether human theory of mind will suspend its attribution of planning in these circumstances.

Below we first provide a summary of research on habitual and planned instrumental behavior. We then report a series of experiments that explore the implications of the plan-habit distinction for theory of mind and moral reasoning.

### Habits and Plans in Instrumental Behavior

Classic work by Dickinson and colleagues [33, 34] argued that planned and habitual strategies constitute two separate systems of behavioral control. The habitual system essentially follows Thorndike’s *law of effect* [35]: an action that leads to reward becomes more likely to be chosen in the future. An influential contemporary operationalization of this law is known as “model-free” reinforcement learning, whereby the value (average reward) of each action is estimated from experience, coupled with a choice policy that probabilistically selects actions in proportion to their value [17]. In contrast, the planning system learns a causal model of the environment (i.e., the reward and transition functions) and uses this model to formulate a plan (hence, it is “model-based”).

Model-free and model-based systems embody different computational trade-offs. The model-free system is computationally efficient, because it caches values in a look-up table, but it is inflexible because it does not make use of a model. Specifically, the model-free system cannot modify its policy in response to changes in the environment unless it directly experiences the long-term consequences of following that policy. The model-based system is computationally inefficient, because it requires some form of expensive tree search or dynamic programming to compute values, but by using a model it is flexible: It can rapidly modify its policy in response to environmental changes without having to experience the policy’s long-term consequences. It has been proposed that early during learning, while the model-free values are still poorly estimated, the model-based system controls behavior, but control shifts over time to the model-free system as its value estimates become more accurate.

This dual-system proposal enjoys considerable empirical support [18, 33, 34]. For illustration, we will describe a study reported by Adams [36]. Rats were first trained to press a lever for food. Next, the food was devalued by pairing it with illness (in the absence of the lever). Finally, the rats were again presented with the lever. Extensively trained rats continued pressing the lever, consistent with a model-free system that is unable to update the value of a lever press following devaluation, since the rat was never exposed to a negatively reinforced lever press. In contrast, moderately trained rats abstained from lever pressing, consistent with a model-based system that controls behavior early during training and can update its internal model of the environment following devaluation.

Several factors are known to influence or covary with the balance between model-based and model-free control. Because the model-based system requires more computationally expensive operations, it has been hypothesized that model-based control should increase with the availability of cognitive resources. For example, placing people under working memory load causes choice behavior to resemble a pure model-free pattern [37], and individual differences in working memory capacity [38, 39] and cognitive control [40] predict the degree of model-based control. Another prediction, confirmed experimentally [37], is that model-based control will increase response times relative to model-free control.

In summary, research on reinforcement learning has established a detailed picture of how plans and habits compete for behavioral control. Although the nature of interactions between these systems is still an active area of research [38, 41, 42], the picture is sufficiently consistent that we can ask: How well do the intuitive and scientific theories of instrumental behavior align?

### Overview of the Experiments

All of our experiments have a similar basic structure. We present participants with information about an agent’s goals and action history, and ask them to make predictions about future actions. The question is whether these action predictions are affected by factors known to govern the balance between model-free and model-based control.

Experiments 1a–c use a simple maze-like navigation task reminiscent of previous research (e.g., [7, 43]). In these experiments, we manipulate an observed agent’s action repetition, a factor which tends to shift control to the model-free system [33]. If participants use this factor to determine whether the agent is model-based or model-free, then they should predict that an agent who has repeatedly taken a particular route will fail to take a novel shortcut.

Experiments 2–4 use vignettes to explore three other factors: consistency of past actions, decision time, and cognitive load. We hypothesized that action predictions would follow model-free policies when past actions were highly consistent, decision time was fast, and cognitive load was high (see [37]). Additionally, we examined the effect of these factors on moral evaluation (i.e., how blameworthy a particular action is). We hypothesized that actions would tend to be less blameworthy to the extent that the model-free system is perceived as controlling behavior.

## Experiment 1a: Manipulating repetition

**Fig 1:**
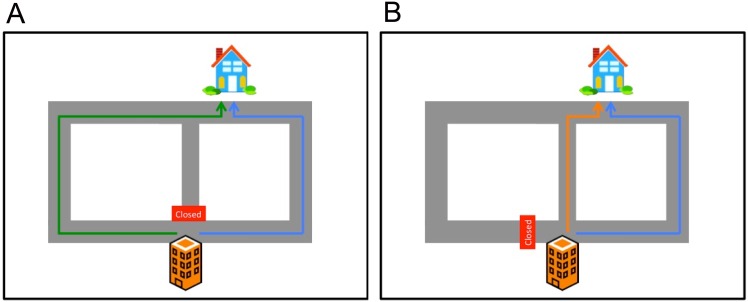
Stimuli from Experiment 1. (A) Participants are asked to predict which path (indicated by blue and green line) the agent will take from work to home. After making a prediction, participants are shown the agent’s chosen path. (B) On the final trial, a shortcut opens up and participants are given a choice between the shortcut (orange line) and the previously preferred path (blue line).

A widely replicated observation in the instrumental learning literature is that repetition promotes the formation of habits (i.e., a shift to model-free control; [33, 36]). In this experiment, we ask participants to make predictions about the actions of an agent while manipulating the number of times that the agent has repeated an action in the past. Specifically, we show participants an aerial view of a simple map and ask them to predict the path that an agent will take from work to home (Fig 1). Some participants observed the agent taking the shortest path twice, while others observed the agent taking the shortest path 8 times. On the last trial of the task, a novel shortcut is opened up, rendering the previously taken path relatively inefficient. The principle of rational action, as embodied by model-based control, stipulates that the agent should take the novel shortcut. However, if participants believe that control has shifted to the model-free system after repeated action, then they should predict that the agent will be relatively more likely to take its previously chosen path in the 8*x* condition compared to the 2*x* condition.

Of course there are several explanations for our hypothesized result other than the influence of a folk theory of habits. People may be more confident in inferring that a person prefers a particular path after observing a greater number of episodes in which it is chosen; people may assume that actors become emotionally attached to that which is familiar; and so forth. While the hypothesized result is not a unique prediction of our theory, it is nonetheless a falsifiable prediction. If people do not infer an increased likelihood of performing an action after repeated reinforcement, this would be strong evidence against spontaneous deployment of a folk theory of habits. Moreover, Experiment 1 establishes a basic effect that we exploit in more nuanced designs to follow.

### Method

#### Participants

264 adults (*N* = 140 for the 8*x* condition and *N* = 124 for the 2*x* conditions) were recruited via the Amazon Mechanical Turk web service [44]. Experiments 1a-c were approved by the Harvard Internal Review Board. All participants received informed consent and were paid for their participation.

#### Stimuli and Procedure

Fig 1 summarizes the structure of the experiment. On each trial, participants predicted which path an agent would take to work using a 6-point Likert scale, and then received feedback about the agent’s chosen path. The agent consistently chose the shorter path (blue line in Fig 1). We varied the number of repetitions of a particular action sequence (either 8*x* or 2*x*) between subjects. On the final trial, participants were given a choice between the repeatedly chosen (habitual) action and a novel shortcut.

After making their prediction on the final trial, participants were given feedback showing that the agent took the habit-consistent action. The participants were then asked to provide a written explanation of why they thought the agent failed to exploit the shortcut.

### Results and Discussion

**Fig 2:**
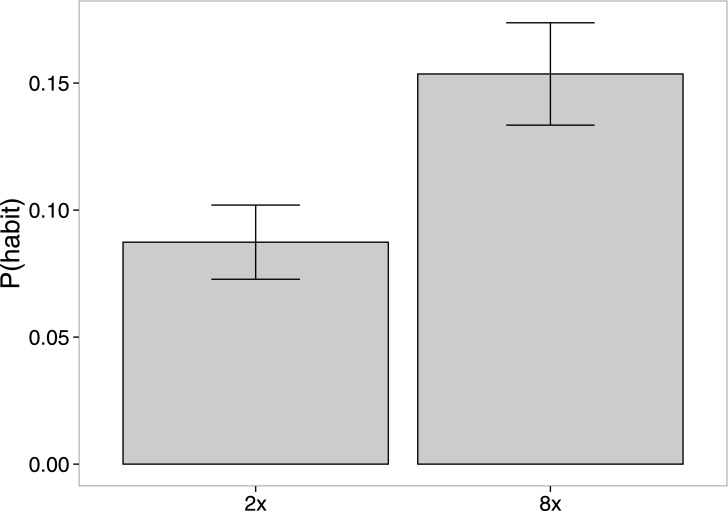
Experiment 1 results. Predicted probability of habit-consistent action (blue path in Fig 1B). Error bars are ±1 *SEM*.

Consistent with the role of repetition in habit formation, participants predicted the habit-consistent behavior more often after observing 8 repetitions, compared with 2 repetitions (*t*(262) = 2.6, *p* < 0.01; Fig 2). Interestingly, while participants were sensitive to the number of repetitions, most participants (94% in the 2*x* condition, 86% in the 8*x* condition) predicted that the agent would take the shortcut—i.e., follow the principle of rational action. While we do not know exactly how quickly habits would emerge in this kind of navigational behavior, studies of virtual maze navigation suggest that after 9 repetitions of a virtual maze, most participants overlooked 50 percent of subsequently available shortcuts [45]. Thus, it appears that participants in our experiment tended to over-apply the principle of rational action, while nonetheless changing their predictions in accordance with the expectation of progressive habitization.

To complement these results, we also analyzed participants’ written explanations of the agent’s failure to exploit the shortcut (i.e., after we stipulated to participants that the agent persisted in taking the old route despite the presence of the shortcut). We recorded the proportion of explanations in each condition that included any of the following words or phrases: habit, routine, regular, used to, usual, always, generally, same way, autopilot, normal, familiar. From an inspection of the responses, these cover the set of explanations that invoke some form of habitual action. We found that habits were invoked by 43% of the participants in the 2*x* condition, and by 57% of the participants in the 8*x* condition. The difference between these two proportions was significant (*p* < 0.05, two-sample binomial test). Thus, written explanations appear to acknowledge that deviation from the principle of rational action may occur when actions are under habitual control, and that this explanation figures more prominently in explanations of behavior following a larger number of repetitions.

We draw two preliminary conclusions from this study: (1) Participants show an overall tendency to predict planning-based action, and (2) increasing the number of repetitions causes subjects to shift from predictions of planning-based action towards predictions of habitual action.

## Experiment 1b: Parametric manipulation of repetition

In Experiment 1b, we sought to replicate and extend the findings of Experiment 1a. Instead of showing participants a series of actions, we asked them to imagine that a path was repeated a certain number of times and then judge the probability that the action would be repeated once the shortcut opened up. This method allowed us to parametrically measure the effect of repetition on action prediction.

It also allowed us to address several potential alternative explanation of our results from Experiment 1a. First, in Experiment 1a, participants may have judged the familiar route more likely simply because they repeatedly predicted—and witnessed—the actor taking that route. This explanation does not apply to Experiment 1b, in which participants are simply informed of the number of prior episodes in which the agent took the familiar route propositionally, rather than witnessing it directly. Moreover, in Experiment 1b we explicitly invoked and queried the habit concept, asking participants how likely it is that the agent’s response will be habitized after *N* repetitions of the familiar route. This method addresses the concern that participants might have predicted that the agent would repeat her behavior in Experiment 1b because of a preference for the familiar, or for any other reason sensitive to the agent’s history other than habituation.

Because Experiment 1b explicitly invokes the habit concept, however, it cannot assess whether this concept is spontaneously deployed (i.e., when not explicitly invoked by the experimenter). In other words, Experiment 1b asks whether the habit concept is sensitive to information We investigate spontaneous deployment of the habit concept in subsequent experiments.

### Method

#### Participants

51 participants were recruited via the Amazon Mechanical Turk web service.

#### Stimuli and Procedure

The stimuli used in Experiment 1b were identical to those used in Experiment 1. In Experiment 1b, participants were given a background story about an agent commuting from work to home: “Bob just moved to a new town, where he commutes each day to work. A map of the town is shown on the right. Bob’s typical route home from work is shown on the map.” Participants were then given the following task: “Now imagine that the shortcut, which was previously closed, is one day opened. Bob has a choice between his typical route (blue line) and the shortcut (orange line). Bob may fail to take the shortcut if he has formed a habit of always taking his typical route. The strength of this habit may change with the number of times Bob has taken his typical route. In the following questions, we will ask you to estimate how long it will take for Bob’s route to become habitual. How likely is it that Bob will take the shortcut after *N* days of commuting?” Here *N* took values of {5, 10, 20, 40, 60, 100}, presented in order of increasing *N*. Each trial was identical, differing only in the value of *N*.

### Results and Discussion

**Fig 3:**
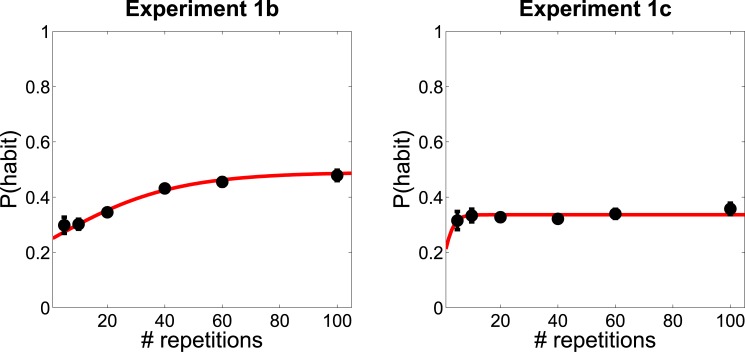
Experiment 1b-c results. Predicted probability of habit-consistent action as a function of number of repetitions (black circles). The red line shows the least-squares fit of a three-parameter exponential model, *y* = *a*/(1 + exp(−b*x − *c*)). (Left) Experiment 1b, in which the habit concept was primed. (Right) Experiment 1c, in which the habit concept was not mentioned explicitly. Error bars represent standard error of the mean.

As shown in Fig 3, the predicted probability of a habit-consistent action increased monotonically with the number of repetitions. To quantify this effect, we computed the Spearman rank correlation between number of repetitions and habit-consistent action probability for each participant. On average, this correlation was 0.57, significantly greater than 0 (*p* < 1*e* − 5, after Fisher z-transforming the correlation coefficients). This confirms the results of Experiment 1, evidencing a parametric shift from planning-based towards habitual action predictions. Once again, we see an overall reluctance to impute habits: The predicted probability of habit-consistent action was significantly less than 0.5 even after 20 repetitions (*t*(50) = 4.54, *p* < 0.0001) and appeared to asymptote around 0.5.

The results of Experiment 1b reveal one discrepancy with Experiment 1a: participants predict a habit-consistent action with probability 0.3 after 5 repetitions while the same probability was 0.15 after 8 repetitions in Experiment 1a. In Experiment 1a, participants may have judged the familiar route more likely simply because they repeatedly predicted—and witnessed—the actor taking that route. This explanation does not apply to Experiment 1b, in which participants are simply informed of the number of prior episodes in which the agent took the familiar route propositionally, rather than witnessing it directly.

## Experiment 1c: Parametric manipulation of repetition without habit priming

Experiment 1b addresses the question,“When we explicitly query observers’ habit concept, is it sensitive to the agent’s history of repetition?” In Experiment 1c, we examined under what conditions observers will apply the habit concept more spontaneously. Thus, we reran Experiment 1b with modified instructions that did not mention habits, and instead asked participants the likelihood that Bob would continue to take the familiar route.

### Method

#### Participants

33 participants were recruited via the Amazon Mechanical Turk web service.

#### Stimuli and Procedure

The stimuli and procedure in Experiment 1c were identical to Experiment 1b, except for the instructions: “Bob just moved to a new town, where he commutes each day to work. A map of the town is shown on the right. Bob’s typical route home from work is shown on the map. Now imagine that the shortcut, which was previously closed, is one day opened. Bob has a choice between his typical route (blue line) and the shortcut (orange line). Bob may fail to take the shortcut and instead repeat his typical route. In the following questions, we will ask you to estimate the probability that Bob fails to take the shortcut after a certain number of days of commuting.” These instructions were designed to omit any mention of habits.

### Results and Discussion

The predicted probability of a habit-consistent action showed a modest increase with the number of repetitions, but much weaker than the results in Experiment 1b, and not statistically significant (Fig 3). The Spearman rank correlation between number of repetitions and habit-consistent action probability was on average 0.06, not significantly different from 0 (*p* = 0.47, after Fisher z-transforming the correlation coefficients). The correlation was significantly smaller in Experiment 1c compared to Experiment 1b [*t*(44) = 2.45, *p* < 0.02]. Thus, the parametric sensitivity to repetition found in Experiment 1b depends on priming a habit concept prior to action prediction.

Summarizing across Experiments 1a-c, we conclude that when participants are prompted to invoke the habit concept, they exhibit sensitivity to the agent’s history of action repetition. In this manner the folk theory of habits conforms to a well-described feature of the actual phenomenon. On the whole, however, participants appear to prefer to predict behavior on the assumption of goal-directed action. Critically, we did not find strong evidence that participants *spontaneously* deploy the habit concept—that is, when it is not explicitly invoked by the experimenter. The apparent spontaneous deployment in Experiment 1a is ambiguous because the results are consistent with a number of alternatives, and our null finding in Experiment 1c provides some evidence against spontaneous deployment. While the reason for this is unclear, we speculate that navigation problems may (at least in the folk theory of mind) preferentially invoke planning.

The remaining experiments are motivated by two goals. First, we aim to provide further tests of the possibility that participants spontaneously deploy the habit concept in some circumstances. Second, we aim to test whether the habit concept embodies other well-described features of the actual phenomenon, beyond its sensitivity to the history of action repetition.

## Experiment 2a: Manipulating learning history

Experiments 2–4 make use of a new scenario, involving an agent who has just started a job at a new company. The agent is warned that there is a problem with the door knob at his new office. The knob works fine as long as it is turned clockwise but gets stuck if it is turned counter-clockwise. In the experiments, we manipulate factors that are known to influence habit formation, as well as factors that influence whether action selection is more likely to be guided by the habitual or the planning system [18]. Specifically, we described situations in which the worker’s door knob at home turns in one direction (potentially establishing a habit), while the new door knobs at work lock when turned in that direction (potentially rendering habitual control maladaptive).

While participants in Experiment 1 were overall somewhat reluctant to infer that the agent’s action selection was influenced by habit, we expected that the scenario used in Experiments 2–4 would be more likely to trigger the inference that the agent’s action was under habitual control. Everyday experience with door knobs suggests that there is variability in the direction that door knobs turn, and this could therefore lead to habit-based errors.

### Methods

#### Participants

121 participants were recruited via Amazon Mechanical Turk. Experiments 2 to 4 were approved by the MIT Institutional Review Board.

#### Stimuli and Procedure

At the beginning of the experiment, participants were informed that the background story would remain the same, but that some additional information about the agent would vary between situations.

In one scenario, the agent’s door knobs at home worked by turning them clockwise, while in the other scenario, the door knobs worked by turning them counter-clockwise. We manipulated this information about the agent’s learning history within participants, and randomized the order in which both scenarios were presented.

**Fig 4:**
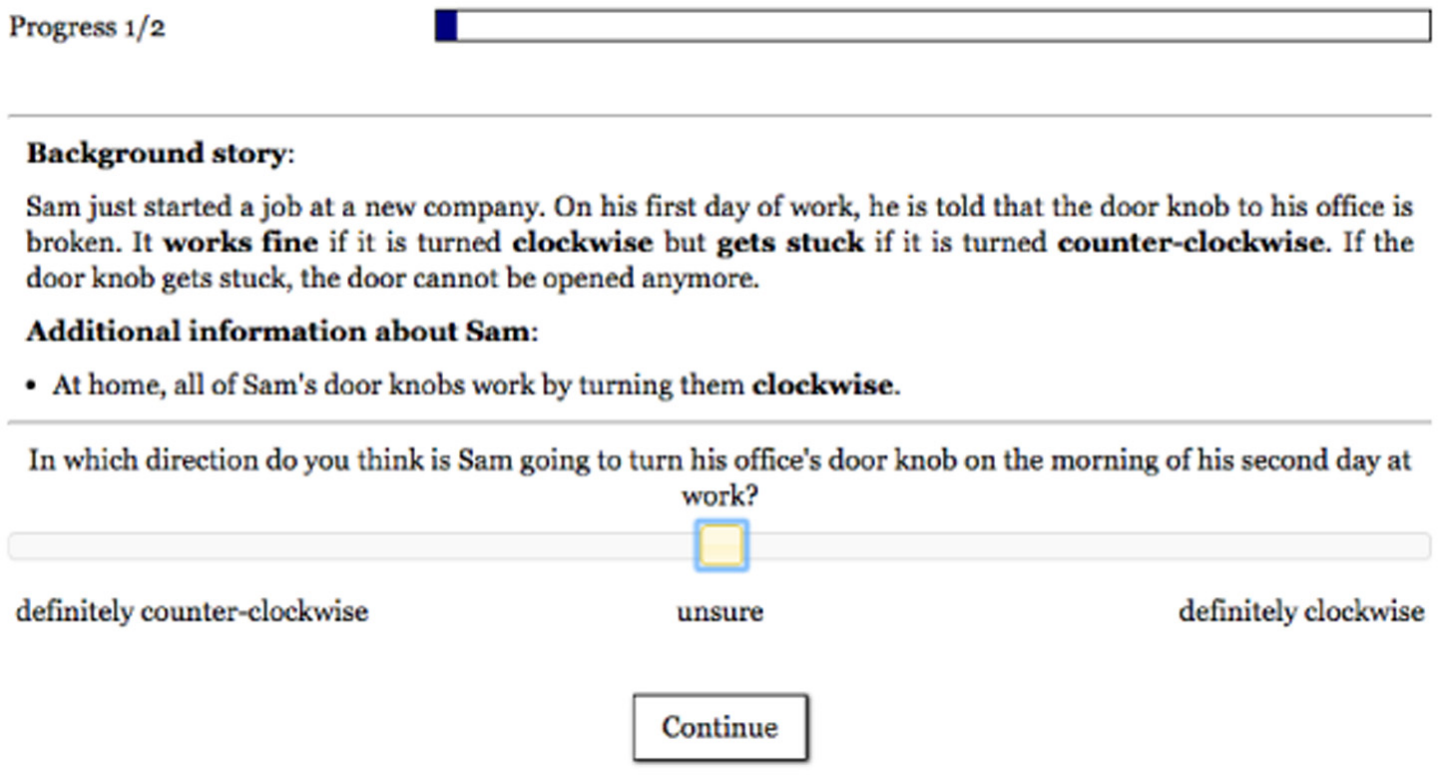
Screenshot of the prediction condition in Experiment 2.

We manipulated what question participants were asked about the scenario. In the *prediction condition* (N = 37), participants were asked to predict in which direction the agent will turn the door knob (see Fig 4). Participants indicated their response on a sliding scale ranging from “definitely counter-clockwise” (0) to “definitely clockwise” (100). The midpoint of the scale was labeled “unsure”.

In the *blame condition* (N = 43), participants read the same background story as in the prediction condition. Again, we manipulated how the agent’s door knobs functioned at home. Additionally, participants read that the agent had actually turned the knob counter-clockwise and that the door got stuck as a result. The agent’s co-worker was trapped in the office for the whole day until the handyman arrived to fix the door. Participants judged to what extent they would blame the agent for the negative outcome by indicating their response on a sliding scale which ranged from “not at all” (0) to “very much” (100).

In the *explanation condition* (N = 41), participants read the same story as in the blame condition. Participants were then asked to explain why the agent had turned the door knob counter-clockwise by selecting one of three response options: (1) “He was used to turning the door knobs this way.” (habit explanation), (2) “It was an accident.” (accident explanation), or (3) “He wanted to trap his co-worker.” (goal-directed explanation). The order of the response options was randomized between participants.

### Results and Discussion

#### Prediction condition

**Fig 5:**
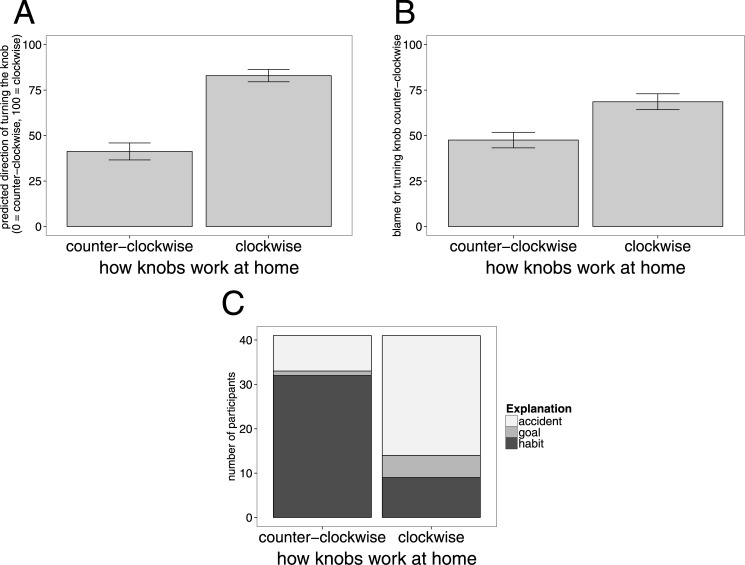
Experiment 2a results. Mean ratings for the prediction condition (A) and blame condition (B), as well as explanations for the agent’s action (C) as a function of how the knobs at home work. Error bars in (A) and (B) are ±1 *SEM*.

Fig 5A shows participants’ judgments about the direction in which they expect the agent to turn the door knob as a function of the agent’s learning history. In general, participants expected the agent to turn the door knob at work in the same direction in which his door knobs at home worked. Participants were more likely to think that the agent would turn the knob in the office clockwise when the door knobs at home worked clockwise (*M* = 82.95, *SD* = 22.39) than when they worked counter-clockwise (*M* = 41.26, *SD* = 30.65, *t*(42) = 7.02, *p* < 1*e* − 7). Importantly, even though the agent was informed that the door would get stuck if he turned the knob counter-clockwise, participants were on average still more likely to think that he would turn the knob in the wrong direction when he was used to turning it this way (*t*(42) = −1.87, *p* = 0.034, one-sided t-test with the midpoint of the scale (50) as the comparison point).

This experiment replicates in a different story context the main effect of learning history on action prediction found in the previous experiments. In contrast to Experiment 1 where participants were reluctant to infer that the agent would take the action that is consistent with the habitual system, we found a much stronger effect of beliefs about habit on participants’ action predictions in this experiment. Participants predicted that the agent’s action would be consistent with having been under habitual control even when it conflicted with his goal (assuming that most agents would rather enter the office than trap their coworker on their first day of work). It is not clear why we find a greater tendency to predict habitual action in the current experiment compared to the previous experiments, but one possibility is that participants have stronger beliefs about habit formation in everyday routine action like turning door knobs compared to spatial navigation (which often tends to be planning-based). Another possibility is that our experimental procedure directed attention towards habitual aspects of behavior. Yet another factor could be the duration of the action sequence. People might be more likely to believe that planning-based behavior can be “overruled” by habitual behavior for short action sequences (turning the door knob) vs. longer action sequences (driving home).

#### Blame condition

Fig 5B shows participants’ blame judgments as a function of how the agent’s door knobs work at home. Participants considered the agent to be more blameworthy for the negative outcome when his door knobs at home worked clockwise (*M* = 68.59, *SD* = 26.61) rather than counter-clockwise (*M* = 47.51, *SD* = 26.03, *t*(36) = 5.35, *p* < 1*e* − 5).

When participants had reasons to believe that the agent may have acted habitually rather than deliberately, they considered him to be less blameworthy for the outcome. That is, when the agent’s door knobs at home all worked counter-clockwise, participants inferred that the agent turned the door knob counter-clockwise not because he wanted to trap his co-worker but rather because he acted out of habit. In contrast, when the agent’s door knobs at home worked clockwise, he had less of an excuse for why he turned the door knob at work in the wrong direction. As one participant put it: “The first guy did it by accident but the second guy seemed to do it just for spite. The first scenario I could understand because he was used to turning the door knobs at home so when he reached for the door knob, he turned it from habit. The second scenario I could not understand how he made a mistake. It almost had to be a deliberate effort to turn the door knob to lock the door.”

Experiment 2 established a close connection between action prediction and blame. Agents whose actions were not easily explained by appeal to habit were deemed more blameworthy for the outcome that their behavior caused. Participants’ action predictions were not solely determined by how a planning-based agent would behave, but were also influenced by their beliefs about whether the agent may have formed a habit.

### Explanation condition

Fig 5C shows the explanations participants selected for why the agent turned the door knob counter-clockwise as a function of how the agent’s door knobs work at home. The proportion of habit and accident explanations differed significantly between the two conditions (*β* = 2.48, *p* < 1*e* − 5). When the agent’s door knobs at home worked counter-clockwise, the majority of participants explained the agent’s action as being the result of habit (32 out of 41). 8 participants explained the agent’s action as being the result of an accident, and only 1 participant said that the agent wanted to trap his co-worker.

In contrast, when the agent’s door knobs at home worked clockwise, the majority of participants thought that the agent’s action was an accident (27 out of 41). 9 participants explained the agent’s behavior as the result of habit (note that this explanation is in fact inconsistent with the agent’s experience in this condition), and 5 participants thought that the agent wanted to trap the co-worker in the office.

How participants explain an agent’s action depends on what they know about the agent’s learning history. When the agent’s suboptimal action was in line with his learning experience, most participants inferred that the agent’s action was habitual. When the suboptimal action was inconsistent with the learning history, most participants inferred that the action must have been accidental. Only a few participants inferred that the agent trapped his co-worker deliberately, and they were more likely to endorse this explanation when the agent’s action was inconsistent with his learning history. Taken together with the results from the blame condition (see Fig 5B), the results of the explanation condition suggest that people are less likely to blame an agent for an action that was inferred to be due to habit compared to an accidental action that may have resulted from the agent’s carelessness.

## Experiment 2b: Explanations and blame

In Experiment 2a, we investigated explanations of behavior and blame for behavior in a between-subjects design. Based on the results, we speculated that in our scenario, people deem negative outcomes more blameworthy when they were the result of an accidental rather than a habitual action. In Experiment 2b, we directly test this hypothesis by asking the same participants to first provide an explanation, and then judge the blameworthiness of the actor.

### Methods

#### Participants

40 participants participants were recruited via the Amazon Mechanical Turk web service.

#### Stimuli and Procedure

The stimuli were the same as in Experiment 2a. The order in which the two scenarios were presented was again randomized. In contrast to Experiment 2a, participants were asked to both select an explanation for the person’s action, and then rate to what extent the agent is to blame for the negative outcome. Participants always selected an explanation before making their blame judgment.

### Results

**Fig 6:**
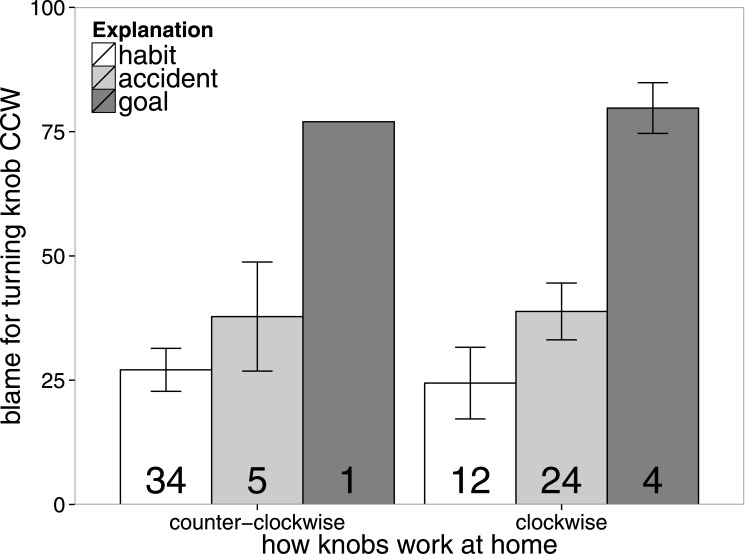
Experiment 2b results. Mean blame ratings separated by condition (x-axis) and by endorsed explanation (different bars). Error bars are ±1 *SEM*. *Note*: The numbers indicate how many participants endorsed each explanation for the two scenarios.

Fig 6 shows participants’ blame judgments as a function of what explanation they selected, separately for each scenario. The pattern of participants’ explanation selections replicates what we found in Experiment 2a. The proportion of participants who explained the agent’s action as habitual versus accidental differed significantly between conditions (*β* = 2.98, *p* <.01). Participants were most likely to explain the agent’s action as having been the result of habit for the scenario in which the agent was used to turning door knobs this way. When the agent’s action was inconsistent with his learning history, most agents believed that the action was accidental.

Participants’ blame judgments also replicate the results from Experiment 2a. The agent was blamed significantly more in the clockwise scenario in which his action was inconsistent with his learning history (*M* = 38.60, *SD* = 29.70) than in the counter-clockwise scenario (*M* = 29.68, *SD* = 25.91, *t*(39) = 2.52, *p* <.016). Overall, blame judgments were lower in Experiment 2b than they were in Experiment 2a.

Finally, blame judgments differed depending on what explanation participants had selected. Participants who believed that the agent’s action was planning-based gave the highest blame ratings. In line with what we had hypothesized based on the results of Experiment 2a, participants held the agent significantly more blameworthy when they explained his action to be the result of an accident rather than habit (*F*(1, 40) = 22.47, *p* < 1*e* − 4). One explanation of this effect is that it makes sense to blame people more for accidents than for habits, because accidents very rarely lead to a positive outcome. In general, we would like other people to be careful and discourage them from acting negligently (which may be a threat to our own well-being at some point). However, habits are not necessarily bad—indeed, the only reason we have habits in the first place is because they (unlike accidents) reliably led to reinforcement in the past. Thus, having good moral habits (e.g. offering our seat to people who need it more without much thinking) is positive and should be encouraged.

Another explanation is that accidental action feels “preventable”. You knew what you were supposed to do, you even did it habitually, and yet still you bungled it. By contrast, in the case of a countervailing habit, there was actually a force pulling you in the suboptimal direction. People may feel that such an error is less preventable, or in any event harder to prevent. Malle’s path model of blame [46] emphasizes this feature of preventability.

## Experiment 3: Manipulating decision time

All experiments so far have probed people’s intuitive theory of habit by manipulating the learning history of the agent whose actions they were asked to evaluate. In Experiments 3 and 4, we use the same background story as in Experiment 2 and manipulate additional factors that are known to influence the probability that the habitual system rather than the deliberate system controls action selection. Individuals who respond faster tend to show greater influence of habitual control [47], and there is some evidence that time pressure increases habitual responding [48]. We hypothesize that participants are more likely to predict that an agent will act habitually when he acts fast rather than slow. Accordingly, in Experiment 3 we manipulate the time the agent took to act in the door knob scenario and investigate how decision time affects action prediction and judgments of blame.

### Method

#### Participants

81 participants were recruited via Amazon Mechanical Turk.

#### Stimuli and Procedure

The background story was identical to the one in Experiment 2 (see Fig 4). In addition to manipulating information about how the agent’s door knobs worked at home, we also manipulated *how* the agent acted.

In the *quick decision* version of the scenario, the agent was described as being late for a meeting and having to rush to get his laptop from his office. He immediately turned the door knob of his office. In the *slow decision* version of the scenario, the agent arrives early at work to have plenty of time to prepare for his first meeting. He goes to his office to get his laptop. He first hesitates and then turns the door knob to his office. Both learning history as well as decision time were manipulated within participants. Whether participants were asked to make an action prediction (*N* = 41), or a blame judgment (*N* = 40) was manipulated between participants. The wording of the questions as well as the response measures were identical to Experiment 2.

### Results and Discussion

#### Prediction condition

**Fig 7:**
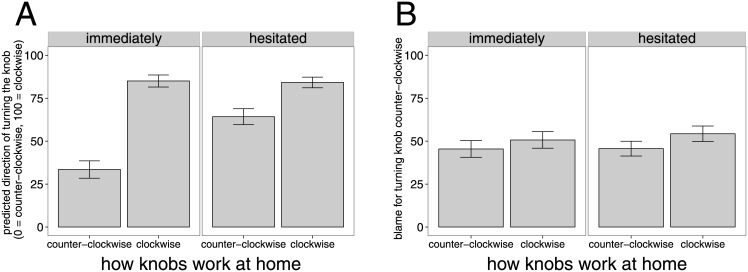
Experiment 3 results. Mean ratings for the prediction condition (A) and blame condition (B) as a function of how the knobs at home work (x-axis) as well as whether the agent acted immediately or hesitated before acting (panels). Error bars are ±1 *SEM*.

Fig 7A shows participants’ predictions about the direction in which the agent will turn the door knob as a function of how the agent’s door knobs work at home, and whether the agent acted immediately or after having hesitated first. There was a main effect of learning history (i.e. how the door knobs work at home; *F*(1, 40) = 68.61, *p* < 1*e*^−^9), a main effect of decision time (*F*(1, 40) = 9.36, *p* <.01), as well as an interaction effect between learning history and decision time (*F*(1, 40) = 17.54, *p* <.001).

In situations in which the agent’s door knobs at home work clockwise, participants expected the agent to turn the knob clockwise no matter whether he acted immediately (*M* = 85.02, *SD* = 22.33) or hesitated first (*M* = 84.17, *SD* = 19.84, *t*(40) = 0.20, *p* = .84). In contrast, when the agent’s door knobs at home work counter-clockwise, participants predicted that the agent will be more likely to turn the door knob at work in the correct direction when he hesitated first (*M* = 64.22, *SD* = 29.80) than we he acted immediately (*M* = 33.49, *SD* = 32.34, *t*(40) = −4.04, *p* <.001).

Participants’ action predictions were both affected by information about the agents learning history as well as by how the agent acted. When the agent acted immediately, participants believed that the agent’s action would be guided by the habitual system. When the agent acted after having hesitated first, participants inferred that the action would be more likely guided by the planning system, even when the agent had acquired a conflicting habit. Comparing the results of this experiment with the previous experiment, we see that participants are even more inclined to believe that the agent’s action will follow the habitual system when he acted immediately. The differences between the action predictions in the counter-clockwise and clockwise condition are significantly greater when the agent acted immediately than in Experiment 2 where no information about decision time was provided (*t*(67.556) = 2.00, *p* <.05).

#### Blame condition

Fig 7B shows participants’ blame judgments as a function of consistency information and decision time. There was a main effect of learning history on participants’ blame judgments (*F*(1, 39) = 11.43, *p* <.001). The agent was blamed more when his learning history was inconsistent with the action (*M* = 52.50, *SD* = 29.31) than when it was consistent (*M* = 45.55, *SD* = 28.76). There was no effect of decision time on participants’ blame judgments (*F*(1, 39) = 0.37, *p* = .55) and no interaction effect (*F*(1, 39) = 0.90, *p* <.35).

Experiment 3 replicates the effect of learning history on blame judgments found in Experiment 2 although the effect was smaller this time. Even though manipulating decision time had a marked effect on action prediction, it did not affect participants’ blame judgments. However, it is important to recall that blame judgments were conditioned upon the observed behavior of the actor, which itself provides an important source of evidence about the influence of habitual control. In other words, although participants thought it unlikely that a person would hesitate and then turn the door knob in the wrong direction, once they observed this event to occur they will likely conclude that it was the product of habitual action (hesitation notwithstanding). This would explain why we observe an effect of decision time on action prediction, and yet not on blame.

## Experiment 4: Manipulating cognitive load

Experiment 3 showed that both learning history and decision time influence action predictions, and that blame judgments are influenced by the agent’s learning history. In this experiment, we manipulate the agent’s cognitive state at the time at which he makes his decision. As discussed in the introduction, placing people under cognitive load leads them to act in a way that is consistent with model-free control [37]. In this experiment, we test whether people’s intuitive theory of how other people make decisions is sensitive to manipulating cognitive load. We predict that people are more likely to think that the agent will act habitually when under high cognitive load than when under low cognitive load. Again, we expect there to be an effect of learning history on participants’ blame judgments. However, given that there was no effect of decision time on participants’ blame judgments in Experiment 3, we refrain from making a prediction about how cognitive load influences attributions of blame.

### Method

#### Participants

81 participants were recruited via the Amazon Mechanical Turk web service.

#### Stimuli and Procedure

The background story was identical to the one used in Experiment 2. In addition to manipulating the agent’s learning history, we manipulated the agent’s cognitive state at the time he took the action. In the *high-load* condition, the agent was described as having a lot of things on his mind. As he turns the door knob to enter his office, he is thinking intensely about the upcoming work meeting with his boss. In the *low-load* condition, the agent was described as looking forward to starting his job. As he turns the door knob to enter his office, he is focused and free from any distracting thoughts. We manipulated learning history and cognitive load within participants, and the type of judgment that participants were asked to make between participants (*N* = 40 in prediction condition, and *N* = 41 in the blame condition). The wording of the questions and the response measures were identical to the ones used in Experiments 2 and 3.

### Results and Discussion

#### Prediction condition

**Fig 8:**
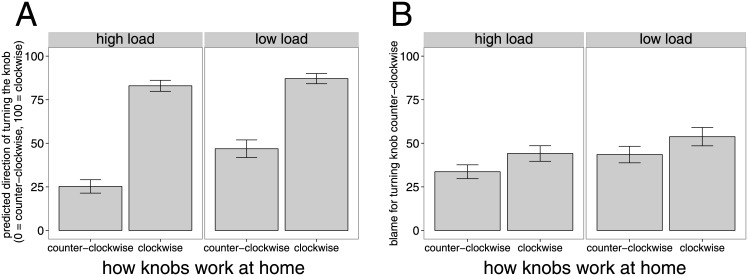
Experiment 4 results. Mean ratings for the prediction condition (A) and blame condition (B) as a function of how the knobs at home work (x-axis) as well as whether the agent was under high or low cognitive load (panels). Error bars are ±1 *SEM*.

Fig 8A shows participants’ predictions about which direction they expect the agent to turn the door knob in the office as a function of the agent’s learning history and of whether he acted under high or low cognitive load. Both learning history and cognitive load significantly affected participants’ predictions (*F*(1, 39) = 100.17, *p* < 1*e* − 11 and *F*(1, 39) = 22.56, *p* < 1*e* − 4, respectively). Participants predicted that the agent will be more likely to turn the knob clockwise when his learning history was consistent (*M* = 92, *SD* = 19.30) rather than inconsistent (*M* = 36.05, *SD* = 30.24). Participants further believed that the agent would be more likely to turn the door knob in the correct direction when he was under low cognitive load (*M* = 67.00, *SD* = 32.89) rather than high load (*M* = 54.10, *SD* = 36.55). There was also an interaction effect between learning history and cognitive load (*F*(1, 39) = 9.28, *p* <.01). Participants predicted that the agent would be particularly likely to turn the door knob in the wrong direction when he had the corresponding learning history and was under high cognitive load.

#### Blame condition

Fig 8B shows participants’ blame judgments as a function of learning history and cognitive load. There was a main effect of learning history (*F*(1, 40) = 8.52, *p* <.01) and of cognitive load (*F*(1, 40) = 12.89, *p* <.001), but no interaction effect (*F*(1, 40) = 0.00, *p* = .97). Participants blamed the agent less when he was used to turning door knobs counter-clockwise (*M* = 38.60, *SD* = 28.12) rather than clockwise (*M* = 48.96, *SD* = 31.44). They blamed an agent more when he had acted wrongly under low cognitive load (*M* = 48.66, *SD* = 32.24) rather than high load (*M* = 38.90, *SD* = 27.32).

The results of Experiment 4 closely mirror the results of Experiment 3. Again learning history affected participants’ action predictions, as well as their assignments of blame. Manipulating cognitive load also influenced action predictions and assignments of blame. Overall, the effects on blame judgments were much smaller than the effects on action prediction. This is consistent with the idea that inferences about whether the person’s action was habitual, accidental or goal-directed is an important aspect of how people assign blame but not the only one. When asked to explain their judgments, many participants argued that the company is in part to blame for not having ensured that all the doors work properly.

## General Discussion

Our studies provide evidence that human action understanding is sensitive to the habit/plan distinction, indicating that our intuitive theory of mind reflects well-characterized features of instrumental learning and control. Experiment 1 showed that, while there is an overall tendency to predict planning-based actions, participants predict that the probability of a habitual response will vary parametrically with the number of times an action has been repeated. As the number of repetitions increases, participants predicted that an agent would tend increasingly to take a habitual route rather than a novel shortcut. Evidence for this parametric response was weaker, however, when it depended upon the participant to spontaneously invoke the habit concept. Subsequent experiments showed that predicted actions were more likely to be habitual when previous actions were highly consistent (Experiment 2), and when action selection occurred under time pressure (Experiment 3) or cognitive load (Experiment 4). Several of these experiments also provide evidence for the spontaneous deployment of the habit concept, without prompting by the experimenter. Furthermore, actions were generally deemed to be less blameworthy when inferred to be under habitual control (Experiment 2).

Our finding that time pressure is diagnostic of habitual control adds a new dimension to a recent line of research on folk interpretations of decision time. Prior research has emphasized that quick (vs. slow) responses are interpreted to reflect high (vs. low) confidence in a decision. For instance, Critcher et al. [49] found that quick decisions are perceived as particularly diagnostic of moral character: An agent who made an immoral decision quickly was evaluated more negatively than an agent who made the same decision slowly, and likewise quick moral decisions were evaluated more positively. When queried about motives underlying the decisions, participants responded that response time reflected the agent’s certainty about moral evaluations. Similar results have been reported outside the moral domain [50]. Thus, humans seem to understand that high-value/high-confidence decisions are made more quickly than low-value/low-confidence decisions, consistent with substantial experimental and theoretical research on decision making [51–53]. The results of Experiment 3 suggest that decision time provides information not only about the value of an option but also about *how* the value is computed: Faster decisions reflect the computations of the habitual system.

On the one hand, it is not surprising that people are able to appropriately deploy the concept of habitual action when explaining, predicting, and evaluating human behavior. People talk about habits and explicitly attribute habitual control to themselves and other routinely. An alternative method to interrogating the folk concept of habitual action would be to simply ask people explicitly what a habit is, how it forms, when it is deployed and how it ought to be morally evaluated. Surely our participants would have provided ready and sensible answers to these questions [5].

On the other hand, several decades of research into mental state attribution converges on the view that people explain behavior in terms of planning [1–6]. The omission of habitual control from this folk conceptual framework is striking, when compared against the prominent role of habit in the scientific framework for understanding the causes of action. One possible explanation for this omission is that, while people possess a concept of habitual action, they tend to fail to deploy it spontaneously as a part of everyday social cognition. By analogy, presumably many physicists with an explicit appreciation of quantum mechanics nevertheless fail to deploy it spontaneously as a part of everyday cognitive engagement with their physical environment. In this case, it would be no more profitable for psychologists to study the ‘folk theory’ of habits than to study the ‘folk theory’ of quantum mechanics.

Motivated by this concern, the studies we present here (except for Experiment 1b) avoid explicitly invoking the notion of habit in the stimuli. Rather, we present individuals with stylized settings modeled on everyday life—situations where habitual control is more or less likely to be exercised—and then test whether their spontaneous behavioral predictions, explanations and evaluations track our manipulations appropriately. We find that they do. We conclude, therefore, that the omission of habitual control from current theories of mental state attribution belies its role as a spontaneously deployed feature of the folk theory of mind.

Our findings raise a host of questions for psychological research. First, do people apportion habitual and planning-based explanations of behavior appropriately—that is, roughly in proportion to the actual occurrence of habitual versus planning-based control exercised by humans [16, 31]? Our present data cannot resolve this question. Although we do estimate of the frequency with which individuals impute habitual control (e.g., when commuting), we have no corresponding estimate of how often a person would act under habitual versus planning-based control in such a circumstance.

Some circumstantial lines of evidence indicate, however, that people may impute habits less often than they should. This would help to explain why concepts of habitual action have played virtually no role in the literature on theory of mind, despite playing a central role in the literature on learning and decision-making. It would also suggest a corollary to the fundamental attribution error (that is, the tendency to attribute behavior to dispositional features of persons rather than situational features of their environment). Specifically, it would suggest that we tend to attribute actions not just to features of a person (vs. their situation) but to features of their *volitional will* in particular (vs. habitual, reflexive, or otherwise automatic causes of action). Of course it remains for future research to test whether people are, in fact, biased to impute planning-based control and, if so, why. One appealing explanation is that people may be introspectively aware of the planning-based causes of their own behavior because planning often demands conscious attention, whereas people may be less frequently aware of the role of habit in causing their behavior because habitual action selection often proceeds without conscious awareness. This idea could be viewed a new twist on the “fundamental attribution error” [54]: people have a tendency not only to explain behavior in terms of personal over situational factors (the classical fundamental attribution error), but also to see an action as generated by a plan rather than a habit.

A second question raised by our study concerns the developmental history of the folk theory of habitual action. Much research assesses when and how children acquire the capacity to represent and infer others’ mental states [55], to predict action based on imputed mental states (e.g., [56–58]), and to subsequently morally evaluate those actions [59–61]. Past research has focused principally on children’s capacity to represent beliefs, also on their capacity to represent desires and goals, and to a lesser degree their capacity to represent emotional states. In contrast, children’s ability to reason about habitual action has been widely neglected.

Although the precise developmental timing varies between tasks [62], a consistent finding is that children find it easier to reason about the actions that an individual will take based on idiosyncratic goals [58] or an unusual environment [57], and more difficult to reason about the actions an individual will take based on their false beliefs [63]. Action prediction based on habitual control presents an interesting intermediate case. A key computational property of habitual action is that it does not depend on an agent’s explicit beliefs (i.e., a causal model). On this basis, we might expect that children would be able to successfully reason about habitual control from an early age. Yet, there is also much evidence that young children have difficulty predicting that others will act in a way that the child themselves knows to be suboptimal or irrational—as when a person acts on false beliefs. Another core computational property of habitual action is that it sometimes leads to locally irrational behaviors (e.g., persisting in taking the old way home in the presence of a new shortcut). On this basis, we might expect that young children would fail to accurately predict behavior on the basis of imputed habits. Evidence suggests children have an apparent a bias to treat inanimate causal agents as if they were animate and sentient [64, 65]—for instance, saying that the clouds make rain because they want plants to grow. This provides additional circumstantial evidence that children may be biased to interpret behavior principally in light of planning, rather than habit.

A third question raised by our research is whether adults’ (or children’s) theory of mind encompasses other control mechanisms, in addition to planning-based and habitual systems. Current theories of instrumental behavior include a third major category: Pavlovian responses [66], which are based on stimulus-outcome associations, independent of action. It remains for future studies to assess whether either or both of these elements are reflected in ordinary and spontaneous reasoning about others’ mental states.

In addition to these psychological matters, our research raises an important question in philosophy and law: How should we assign moral responsibility to actions under habitual control? Here, again, there is surprisingly little past discussion in light of the central role for habitual action posited in the literature on instrumental learning and decision-making. Rather, the default model of behavioral control assumed in the Anglo-American legal tradition is planning based on beliefs and desires [67], and the most frequently invoked alternative form of control is “impulsive” behavior (e.g., a violent act committed upon discovering one’s lover *in flagrante delicto*). To the extent that legal precedent establishes a standard treatment of habitual action, it has been to include it as a form of ordinary “voluntary” control—i.e., exposing the individual who commits a harm out of habit to full legal liability [68, 69]. The results of the present study indicate that, in this respect, legal precedent is out of step with ordinary folk judgments of moral responsibility, since we observed diminished blame for harms committed out of habit.

Philosophers have devoted relatively more attention to the concept of habitual control and its implications for moral responsibility (e.g., [70]), dating back at least as far as Aristotle’s *Nicomachean Ethics*. Contemporary philosophical treatments have argued both for [71] and against [72] assigning moral responsibility to actions performed under habitual control. Future philosophical treatments will surely profit from psychological and neuroscientific research into the actual mechanisms of habitual control; they may also profit from a detailed account of the folk theory of habitual action, specifying the respects in which it aligns and diverges from the relevant scientific theories.

The conceptualization of habit that we have adopted in this work stems from a long “associationist” tradition originating with William James [73], which views habits as arising from an associative learning process. The end result is an inflexible “routine” that can be executed without goal-directed, deliberative control. This conceptualization strongly influenced the animal learning theory perspective articulated by Dickinson [33] and its modern computational formalization [17, 18]. There exist richer conceptualizations of habits that apply primarily to humans [16, 74], but a full consideration of these alternatives is beyond the scope of this paper, because our experiments do not take a strong stand on various fine-grained differences between competing accounts of habitual action. All the models agree on the basic features we test here. An interesting avenue for future work would be to consider experiments that investigate the roles played by different notions of habit in the folk theory of mind.

One issue that we have glossed over is context-dependence: it’s possible that participants have habits as well as internal models that vary with context, and this accounts for some of the patterns in our data. For example, context-dependence, like habitual action, is also sensitive to repetition [75]. Interestingly, recent work suggests that habits are more context-dependent than plans [76]. These issues are not systematically addressed in the present work, where we held context changes constant across conditions. Also, our scenarios stress that the protagonist only just started their job; hence, it is unlikely that they have already developed a context-specific habit at work.

In summary, we have shown that ordinary adults possess a theory of habitual action and spontaneously deploy it in order to describe, explain and predict human behavior. Our research indicates that the folk theory of habits mirrors several key features of contemporary scientific and computational models of habitual control. Specifically, people are more likely to impute habitual control to behaviors that have been frequently repeated, and under conditions of time pressure and cognitive load. Finally, we show that people incorporate their inferences about habitual versus planning-based control in moral judgment, partially (although not fully) excusing harmful actions arising from habit. Although the literature on “theory of mind” in humans has provided a rich, detailed and well-supported characterization of inferences about planning-based action, our initial studies suggest that much work remains in characterizing inferences about other forms of instrumental control.

## Supporting Information

S1 File Experimental data. (ZIP)
